# Printed Smart Devices on Cellulose-Based Materials by means of Aerosol-Jet Printing and Photonic Curing

**DOI:** 10.3390/s20030841

**Published:** 2020-02-04

**Authors:** Mauro Serpelloni, Edoardo Cantù, Michela Borghetti, Emilio Sardini

**Affiliations:** Department of Information Engineering, University of Brescia, Via Branze 38, 25123 Brescia, Italy; michela.borghetti@unibs.it (M.B.); emilio.sardini@unibs.it (E.S.)

**Keywords:** printed electronics, paper-based circuit, paper-based hybrid circuit, aerosol jet printing, photonic curing

## Abstract

Printed electronics is an expanding research field that can reach the goal of reducing the environmental impact on electronics exploiting renewable and biodegradable materials, like paper. In our work, we designed and tested a new method for fabricating hybrid smart devices on cellulose substrates by aerosol jet printing (AJP) and photonic curing, also known as flash lamp annealing (FLA), capable to cure low temperature materials without any damage. Three different cellulose-based materials (chromatographic paper, photopaper, cardboard) were tested. Multilayer capability and SMDs (surface mount devices) interconnections are possible permitting high flexibility in the fabrication process. Electrical and geometrical tests were performed to analyze the behavior of printed samples. Resulted resistivities are 26.3 × 10^−8^ Ω⋅m on chromatographic paper, 22.3 × 10^−8^ Ω⋅m on photopaper and 13.1 × 10^−8^ Ω⋅m on cardboard. Profilometer and optical microscope evaluations were performed to state deposition quality and penetration of the ink in cellulose materials (thicknesses equal to 24.9, 28.5, and 51 μm respectively for chromatographic paper, photopaper, and cardboard). Furthermore, bending (only chromatographic paper did not reach the break-up) and damp environment tests (no significant variations in resistance) where performed. A final prototype of a complete functioning multilayer smart devices on cellulose 3D-substrate is shown, characterized by multilayers, capacitive sensors, SMDs interconnections.

## 1. Introduction

Paper, considered as a substrate for printed electronics, is gaining great attention in the research field because of simplicity of use, together with flexibility and lightweight. Paper substrates are made of multiple layers realizing an inner network of cellulose fibers, the principal component of the plant cell wall, and the most abundant and widespread biopolymer. The reason of the interest in paper materials is related to its unique combination of properties, like biodegradability, biocompatibility, and renewability, thus reducing waste production, important aspects in an environmental-friendly manufacturing context [1]. Paper-based substrates show an intrinsic versatility since different kind of paper can be used as suitable materials depending on the application, from printer paper [2,3], glossy brochure paper, newspaper, cardboard [4] to photopaper [5], and chromatographic paper [6]. Being a disposable material, paper-based sensors have proven their validity in studies of biological samples in laboratories, but they can show their usefulness in resource-limited situations owing to low-cost, robustness and ease-of-disposal, simply burning the device in presence of biological samples avoiding sterilization [7,8]. It is also possible to find their use in other sectors, like environmental monitoring, food and cosmetics industries [9,10].

Paper electronics is emerging owing to the possibility to realize circuits and electrical components avoiding conventional techniques [11,12,13,14] but presenting limitations in the realization of complex devices which can be produced only considering multilayer steps, thus lengthening the manufacturing process [1]. These limitations are usually related to the technologies employed in the realization of such devices, like screen-printing or inkjet printing, which allow only planar printings [11,15,16,17,18,19,20,21,22]. Another alternative can be the so-called 3D-origami technique, consists of a specific paper folding to develop a final electro-device [23,24]. The advantage of this technique is at the same time a drawback and a limitation, since origami electronics devices are simple to use and develop, but they need human presence to follow the specific folding procedure, otherwise the device will be useless. Furthermore, it is not possible to use every type of robot in the bending process, due to both hardware and software problems (mobility and manipulation capabilities on one side, lack of an algorithm capable of manipulating the paper in the correct sequence with the least number of steps on the other side) [25]. Another emerging technique for paper electronics fabrication is transfer printing. Transfer printing method involves the use of an intermediate stamp for the transfer of a geometry on the substrate, obtaining flexible and stretchable complex structures. The main challenge is the ink transfer from the stamp to the final substrate, especially for soft and flexible substrates such as paper [26]. Among this wide family of techniques, laser-assisted printing is very suitable to produce complex geometries, but the heat source must be controlled to avoid defects and damages [27]. In fact, the laser used for sintering the deposited film induces overheating (a few hundred °C) of the substrate, and thus it is not suitable for temperature-sensitive materials such as paper [28].

Considering the abovementioned limitations and other drawbacks typical of printing electronics (limited inks viscosity, nozzle’s clogging, coffee-ring effect of the droplets), aerosol jet printing (AJP) is a viable technique that can outgo these factors [29,30]. AJP has started attracting great attention because of the possibility to print various inks on a wide range of substrates (viscosity range = 1:1000 cP in case of pneumatic atomizer, 1:5 cP in case of ultrasonic atomizer), with a significant reduction in the amount of wasted ink. Furthermore, small droplets dimensions (about 1–5 μm in diameter) and performances achievable (width range = 10 μm–3 mm; thickness = 100 nm–10 μm) are other unique features of this technology [30,31,32,33]. Selective deposition is ensured both on planar and 3D structures, without using masks or post-patterning, and realizing specific surface features. AJP is a full-additive, contactless printing manufacturing process composed of four key steps: Atomization of a liquid suspension by pneumatic or ultrasonic atomization thanks to a carrier gas (nitrogen or compressed dry air); generation of a mist of droplets (around 1–5 μm in diameter) passing through a virtual impactor to remove carrier gas and select droplets dimensions; focus of the stream by a sheath gas; deposition of the atomized ink on the substrate. This 3D printing technique was involved in the development of many applications, like high-efficiency solar and fuel cells, fully printed thin-film transistors, embedded resistors, antennas, MEMS, flexible displays and circuitry [33], photodetectors [34], wearable applications [35], thermistors [36], microelectrodes arrays for biosensing applications [37], lab-on-chip devices [38], protein [39] and glucose sensing [40].

A sintering/curing step is usually requested by manufacturers of printing electronics inks, which is typically performed in oven and becoming problematic for low (melting or firing) temperature materials like cellulose-based. Alternative techniques were raised in order to cover these issues, like photonic curing [41]. This technology, also known as flash lamp annealing (FLA) or intense pulsed light (IPL), was developed by Novacentrix [42], and consists of a couple of mirrors and xenon flash lamps radiating energy toward the sample, following phenomenon of melting/sintering point depression in nanoparticles described by the Gibbs–Thomson equation [43]. The voltage crossing the lamp and the single impulse time duration are the key parameters, responsible of the penetration of the light wave. The advantages are remarkable: the heat treatment takes place in milliseconds, producing minimal damage to low-temperature substrates and avoiding unwanted processes (diffusion) [42]. FLA was employed in different kind of applications ranging from thin-film transistors [44], solar cells [45], antennas [46], RFID components [47,48] to wearable devices [49], using conductive, bioresorbable or ceramic inks (silicon, silver, zinc) on different kind of substrates (wood, cardboard, textiles, glass, polymers) [50,51].

In light of this, the aim of this work is to test the possibility to realize smart devices (also on 3D surfaces and with 3D interconnections and multilayer features) by means of AJP and FLA on cellulose-based substrates, providing a step-by-step guide to fabricate a complete device. The manufacturing method is tested on three different cellulose substrates: chromatographic paper, photographic paper, and cardboard. Printed samples were initially tested to evaluate curing parameters and mechanical behavior in bending situations, together with electrical resistance in presence of a damp environment. Finally, the manufacturing method was used for the fabrication of a multilayer smart-3d-device equipped with printed capacitive touch sensors, electronic components, and 3D interconnections. The proposed method could develop in the future 3D-functionalized objects with low-cost and environmental-friendly materials like cellulose-based one, i.e., objects able to interact with human beings and the environment and measure physical quantities.

## 2. Materials and Methods

### 2.1. Materials

HPS 108-AE1 (Novacentrix, Austin, TX, USA) is the selected conductive ink for printing tracks and interconnections in this work. It is an aqueous suspension of silver flakes, specifically formulated for AJP, containing a polymeric additive to strengthen the adhesion to substrates. For bonding and insulating, a UV-curable polymer is selected, its commercial name is NOA 81 (Norland Products Inc., Cranbury, NJ, USA); it is a fast UV-curing adhesive, which produces, after curing, a hard, resilient bond. This ink is characterized by a viscosity of 300 cP at 25 °C, showing an excellent adhesion on glass, paper, and metal, and a fair adhesion on plastics; it is known to be biocompatible. In this work, it was used as an insulating layer to realize a multilayer structure, as gluing medium to place surface mount devices (SMDs) and as insulator for the capacitive touch sensors developed in the final prototype.

As cellulose substrates, three different types were selected: chromatographic paper, photographic paper, and packaging cardboard. Whatman chromatographic paper grade 1 (GE Healthcare, Little Chalfont, Buckinghamshire, UK), was purchased from Carlo Erba, selected because of its characteristics of purity without additives of any kind that guarantees the total absence of contamination, high quality, and homogeneity. It is used for biosensors, chemical sensors, point of care systems and is characterized by a thickness of about 180 μm. Photographic paper Hp Everyday A4 Photo Paper (Hp, Palo Alto, CA, USA) was purchased from a local shop, selected because of its ease of handling and usage in relationship with inks. Photopaper presents a thickness of about 400 μm. It is widespread and used for low-cost applications. Packaging cardboard (thickness of about 250 μm) was sourced from internal stock and was selected since it is a recycle material, used for packaging thus with a higher mechanical stiffness with respect to the other two selected substrates.

### 2.2. Manufacturing of Conductive Tracks and Insulating Coatings

**Figure 1:**
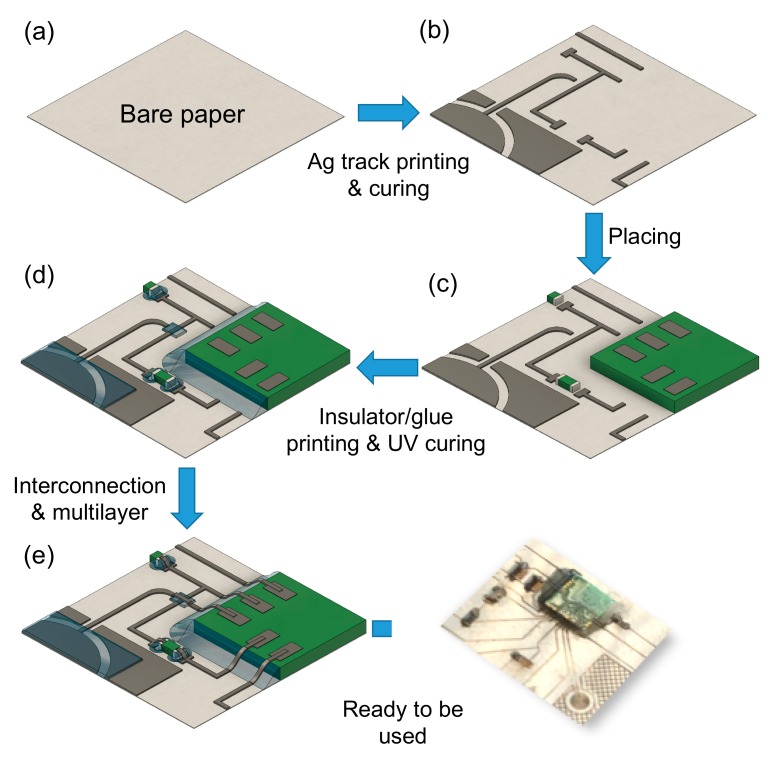
Block diagram of the circuit manufacturing steps: (**a**) bare paper; the first phase (**b**) regards Ag ink deposition and FLA curing; the second step (**c**) involves the placing of surface mount devices (SMDs) and packaged components; step three is focused on (**d**) the deposition of NOA 81 to fix previously placed packaged components and as insulator on specific Ag tracks and capacitive sensors; the last phase (**e**) regards the final Ag deposition in multilayer zones of the circuit and to interconnect Ag tracks with packaged components.

The block diagram in Figure 1 depicts the proposed manufacturing process. In (b), Ag tracks are realized by using AJP and cured by FLA. In (c), SMDs are placed on the substrate. Flat no-leads packages such as quad-flat no-leads (QFN) and dual-flat no-leads (DFN) are placed in a lateral chip configuration, so as to have the pads positioned up. In (d), NOA 81 was selectively deposited by using APJ and exposed to UV light during printing to fix SMDs on cellulose and to make an insulating coating on tracks for multilayer purposes. Phase (e) involves printing Ag track by AJP and curing by FLA to interconnect components. To manufacture multilayer circuits with more than two layers, phase (d) and (e) could be repeated.

#### 2.2.1. Ag ink Deposition and Photonic Curing

**Figure 2:**
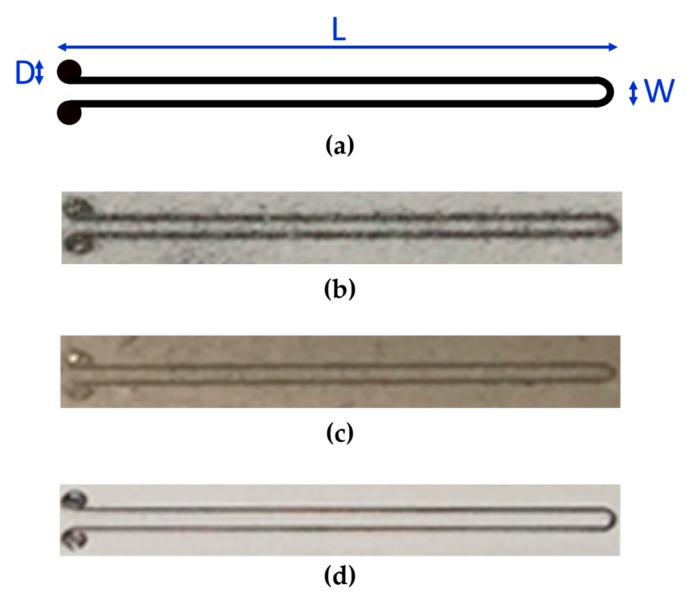
(**a**) Pattern and geometrical dimensions of printed samples (L = 22.5 mm, D = 1 mm, W = 1 mm). Image of resulting samples printed on (**b**) chromatographic paper, (**c**) cardboard, and (**d**) photopaper.

The process parameters considered during the steps regarding Ag ink printing are reported in Table 1. These parameters were tested by an aerosol jet printer AJ300 (Optomec, Albuquerque, NM, USA) employed in this work. To reach the desired conductivity values, Ag ink was deposited ten times on chromatographic paper and cardboard, five times on photographic paper before curing. Figure 2 shows the printed samples that were used for experimental tests on the different substrates. These consist of a U-shaped trace of about 44.55 mm long. After printing Ag ink, the samples were oven-dried at 140 °C as suggested by the ink manufacturer.

Pulseforge 3300 (Novacentrix, Austin, TX, USA) is the FLA-employed machine. Voltage of the lamp and time duration of single impulse are the two main process parameters that were considered in the experiments. To better understand the influence of these two aspects and define the best photonic curing parameters for each substrate, the printed samples were cured with a single lamp flash at four different time durations (1200, 1400, 1600, and 1800 μs) and for each of these, the voltage was varied, increasing this factor of 20 V per flash irradiation. A total amount of 285 samples was tested.

**Figure 3:**
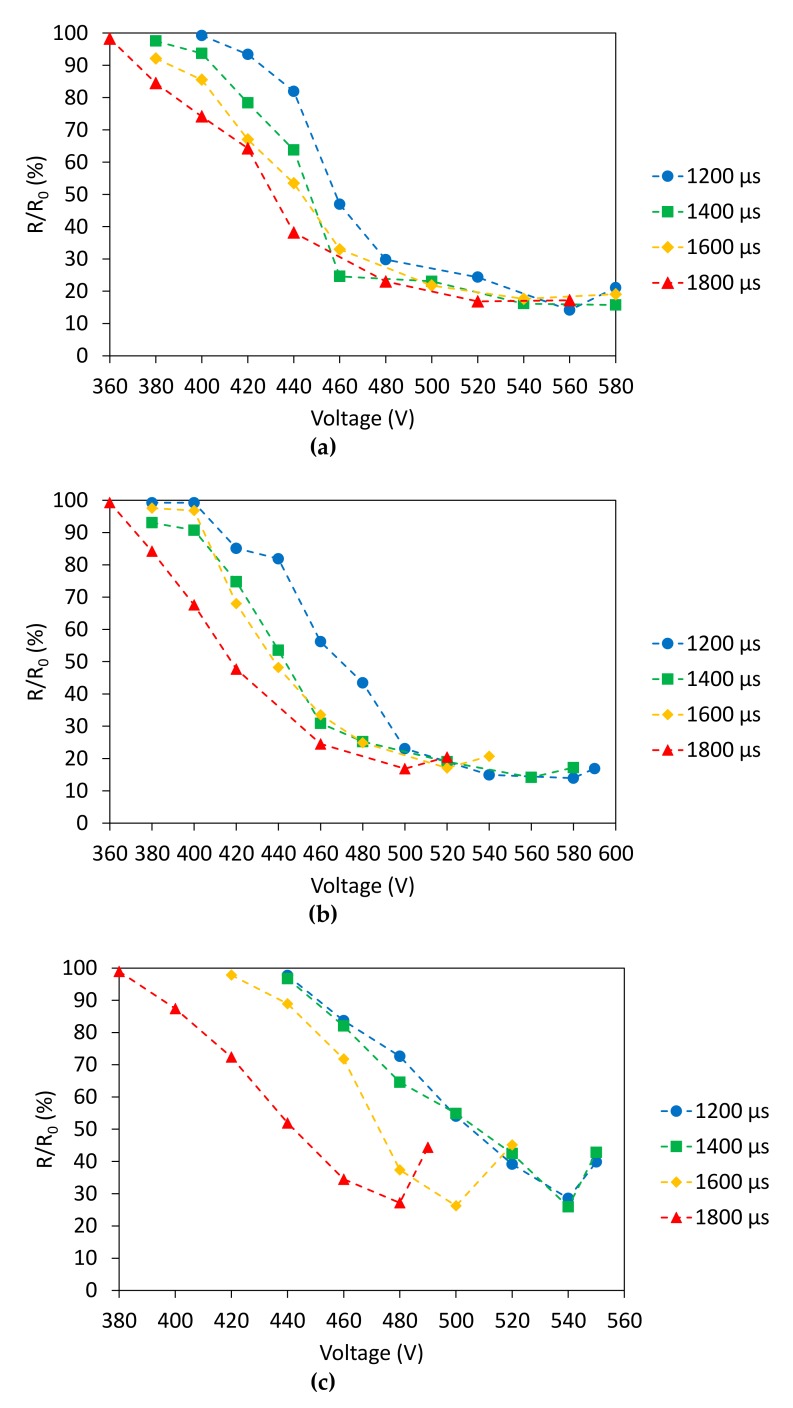
R/R_0_ vs. voltage depending on impulse duration for (**a**) chromatographic paper, (**b**) cardboard, and (**c**) photopaper.

Figure 3 summarizes electrical resistance analysis, performed right before and after curing through the digital bench-top multimeter Hewlett-Packard HP34401a. Testing probes to the pads at the extremities of each sample in 4-wire resistance measurements were applied using an Everbeing probe station with 4 probe holders. Resistance measurements were performed in laboratory under controlled environmental conditions. For each point of the plots in Figure 3, three samples were tested. The results of these measurements are reported in terms of ratio between the final resistance (R) of the sample after curing versus its own resistance before curing (R_0_).

On chromatographic paper, a mean resistance before curing of 11.9 Ω was found, with a relative standard deviation (RSD) of 38%. In Figure 3, each one of the four series shows a significant decrease of the resistance after curing near 460 V, which then deviates in a sort of plateau. The results show a minimum resistance in correspondence of 580 V and 1200 μs, 560 V and 1400 μs, 520 V and 1600 μs, and 500 V and 1800 μs with values of 2.62 Ω (RSD = 9%), 1.7 Ω (RSD = 1%), 1.75 Ω (RSD = 2%), and 1.52 Ω (RSD = 3%), respectively. The last combination (520 V and 1800 μs) appears to be the most efficient, achieving the lowest resistance (1.52 Ω, RSD = 3%), with a resistance reduction of 83%.

Ag samples on cardboard have a resistance before curing of 13.02 Ω (RSD = 19%). The procedure was similar to the one for chromatographic paper substrate, the samples presented a plateau that is common to all sintering durations. More specifically, 1200, 1400, 1600, and 1800 μs, showed their minimum in resistance in correspondence of 580, 560, 520, and 500 V with a value of 1.98 Ω (RSD = 1%), 1.92 Ω (RSD = 19%), 2.085 Ω (RSD = 2%), and 2.085 Ω (RSD = 1%), respectively. The curing process allowed to reach a resistance reduction of about 85%. In order to limit R.S.D., 1200 μs and 580 V can be proposed as best sintering parameters.

Ag samples printed on photographic paper presented a mean resistance before curing of 6.9 Ω (RSD = 39%). Because of the sintering process, it is possible to achieve a resistance reduction of about 73% showing the best compromise in presence of 1400 μs impulse duration at 540 V (mean resistance = 1.26 Ω, RSD = 6%). 1200, 1600, and 1800 μs show their best in correspondence of 540, 500, and 480 V and the specific resistance values are 1.5 Ω (RSD = 10%), 1.78 Ω (RSD = 8%), and 2.035 Ω (RSD = 11%), respectively. In fact, in case of 1600 and 1800 μs, resistance values are higher, and this fact can be connected to a higher thermal stress, which can cause fracture along the length of the sample. This event happened in all situations when the tracks reached a thermal state in which some cracks take place, as witnessed by the ascents shown from each curve at high voltage levels.

#### 2.2.2. Insulator Printing and UV Polymerization

**Figure 4:**
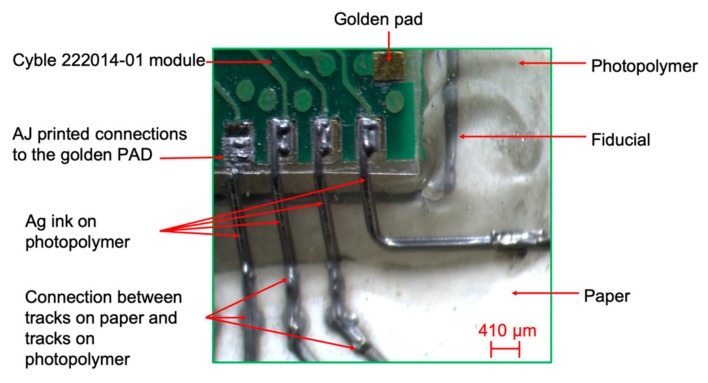
Details of encapsulated programming module and printed tracks.

The capability of AJP to selectively print different inks was investigated, printing insulating or adhesive inks for insulating tracks or fixing SMDs. NOA 81 (Norland Products Inc., Cranbury, New Jersey, USA) is a liquid adhesive photopolymer that can be easily printed by AJP. The ink has been UV-cured during deposition by using the LED Spot type Panasonic ANUJ6180 (Panasonic, Osaka, Japan) and the lens ANUJ6423 as UV Curing System, characterized by a spot diameter of 3 mm, a peak UV intensity of 17,200 mW∙cm^−2^ at an irradiation distance of 8 mm. In our tests, the selected power was 5% of the maximum peak intensity. NOA 81’s specific printing process parameters are reported in Table 2. Specifically, NOA 81 was used as insulating layer for capacitive sensors, to glue SMDs on cellulose-based substrates during placing step and to create the intermediate layer in multilayer zones of the circuit. The SMDs and IC were placed and fixed on the substrates owing to NOA 81, realizing an oblique wall on which connections between component pads and silver tracks on paper were printed. Correct placing was ensured by pads and fiducials, specifically designed for each kind of SMDs to place, as shown in Figure 4.

#### 2.2.3. Multilayer and Interconnections

**Figure 5:**
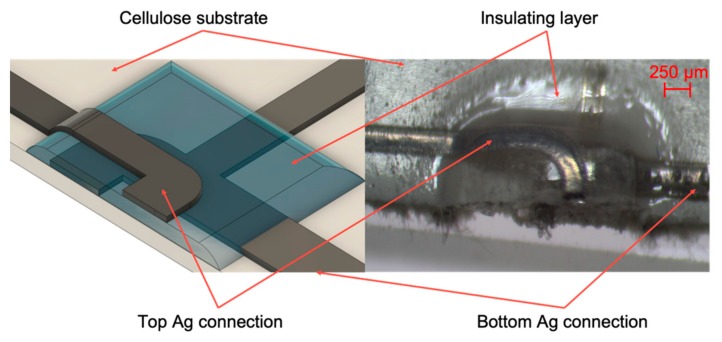
Microscope magnification of a multilayer zone.

Ag ink was selectively printed to interconnects SMDs and previously printed tracks, as shown in Figure 4, or multilayer zones as evidenced in Figure 5, permitting the manufacturing of two or more circuit layers. The optical microscope by Orma Scientific NB50T (trinocular zoom 0.8x–5x–LED), with its devoted HDMI MDH5 camera model, was used to acquire the images (Orma Scientific, Sesto San Giovanni, Milan, Italy).

## 3. Results

### 3.1. Resistivity Analysis

**Figure 6:**
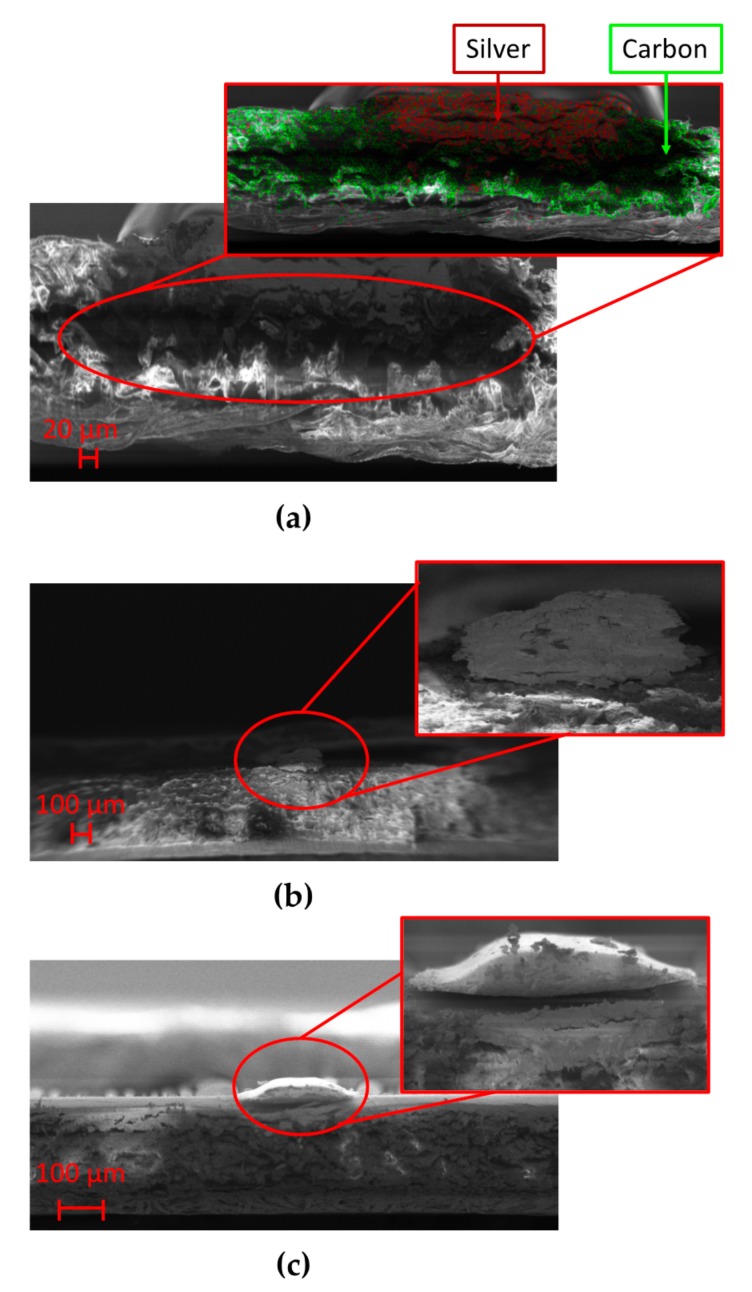
Cross-section magnification performed thanks to the abovementioned field emission scanning electron microscope (FE-SEM) for samples printed on (**a**) chromatographic paper, (**b**) cardboard, and (**c**) photopaper.

In order to calculate the resistivity of the printed samples, geometrical analysis of the tracks was necessary. Figure 6 presents the morphology of the sample cross-sections that was investigated by using a field emission scanning electron microscope (FE-SEM model LEO 1525, ZEISS) operated at 2–10 keV energy beam. The microscope was coupled with an Oxford energy dispersive X-ray analysis (EDX). Samples were attached with carbon glue to metallic stubs to reduce charging effects due to the electron beam. Among the substrate employed in this study, chromatographic paper is the most hygroscopic one with great absorption capacity. This behavior is supported by SEM analysis, Figure 6a shows the cross-section of the track analyzed by the EDX of the SEM system, evidencing the chemical composition of the sample and confirming the depth penetration of Ag ink in chromatographic paper. Thus, considering what stated in the previous chapter, a longer impulse duration to fully penetrate in paper thickness is mandatory.

The need not to involve the entire thickness of the cardboard in the sintering process can be explained observing Figure 6b; the silver ink does not penetrate completely due to its more compact structure, compared to the chromatographic paper that makes porosity its strength. Looking at the photopaper magnification in Figure 6c, one can note that, unlike what happened in the presence of chromatographic paper, Ag ink did not reach the total depth involving only a portion of the substrate thickness, so that high values of impulse duration are not mandatory.

The geometrical analysis was then performed on cellulose samples owing to Filmetrics Profilm 3D optical profilometer (Filmetrics Inc., 10655 Roselle St., San Diego, CA, USA), to evaluate the shape of the printed lines. It is based on state-of-the-art white light interferometry (WLI), a non-contact optical method for surface height measurements on 3-D structures, to measure surface profiles and roughness down to 0.05 µm. The instrument works in the range of 50 nm–10 mm with substrates and materials characterized by a reflectance between 0.05–100%. The system implements a 5MP camera, the Nikon CF IC Epi Plan 20× model (field-of-view: 1.0 mm × 0.85 mm). The samples were measured in three different areas along the total length in order to state the stability of the thickness. The parameters evaluated in this phase are the total thickness, calculated as the difference between the maximum height and 1% of this value, and line width, calculated as difference between two consecutive 1% values on the two sides of the maximum height.

**Figure 7:**
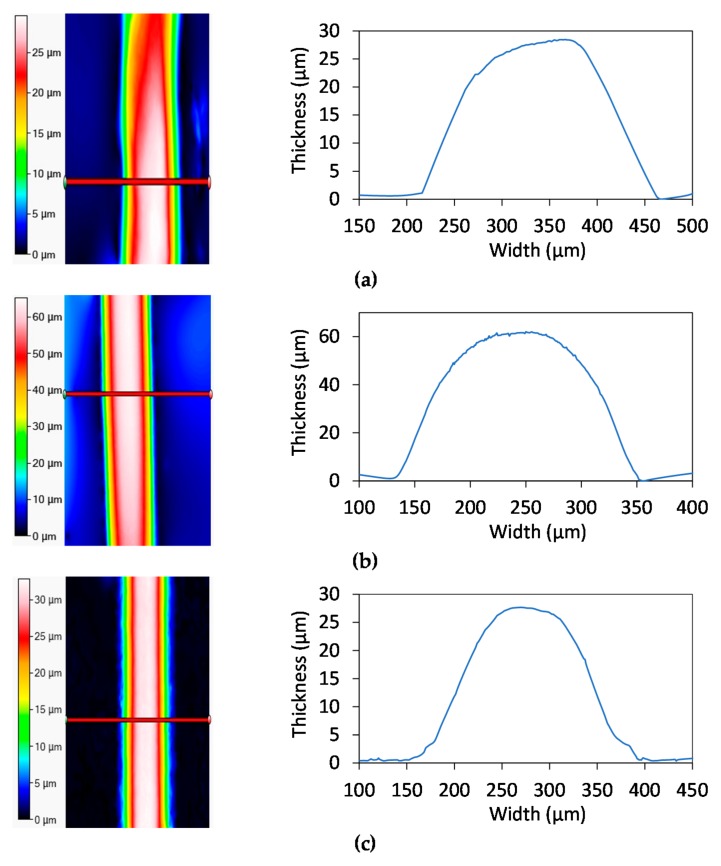
Thickness profile for Ag on (**a**) chromatographic paper, (**b**) cardboard, and (**c**) photographic paper.

Figure 7 shows profilometer data obtained for the three different substrates, with a visual representation of lines geometry and a heat map regarding the thickness of a single line. The mean thickness and line width of the chromatographic samples are, respectively, 24.9 μm and 274 μm with RSD = 15%. The result achieved is due to the intrinsic nature of chromatographic paper, which is a 3D network of fibers with hydrophilic properties which tends to absorb a liquid. Silver deposition on cardboard shows a peak height that is higher respect to chromatographic paper, about 51 μm with a total width about 258.05 μm (relative standard deviation of 18% and 10%, respectively). As for photopaper, the mean thickness for Ag lines printed on this substrate is 28.5 μm with a relative standard deviation of 12%. On the other side, mean line width is 255.5 μm with a relative standard deviation of 4%. It must be kept in mind that the number of depositions on photopaper are the half of the other substrates.

Once defined the geometrical profiles, samples volume resistivity was evaluated and compared with the one reported in the manufacturer data sheet. Volume resistivity was calculated considering the definition of resistance:

R = ρ⋅(l/S)(1)

where R is the total resistance of the sample, l is the total length of the sample and S represents the section of printed track. The calculated resistivity is about 26.3 × 10^−8^ Ω⋅m, 22.3 × 10^−8^ Ω⋅m, and 13.1 × 10^−8^ Ω⋅m respectively for chromatographic paper, photopaper, and cardboard. These values are in agreement with what Novacentrix declared on the data sheet, which are in the range from 14 × 10^−8^ Ω⋅m to 44 × 10^−8^ Ω⋅m, depending on deposition and curing parameters.

### 3.2. Electrical and Mechanical Stability under Bending Conditions

**Figure 8:**
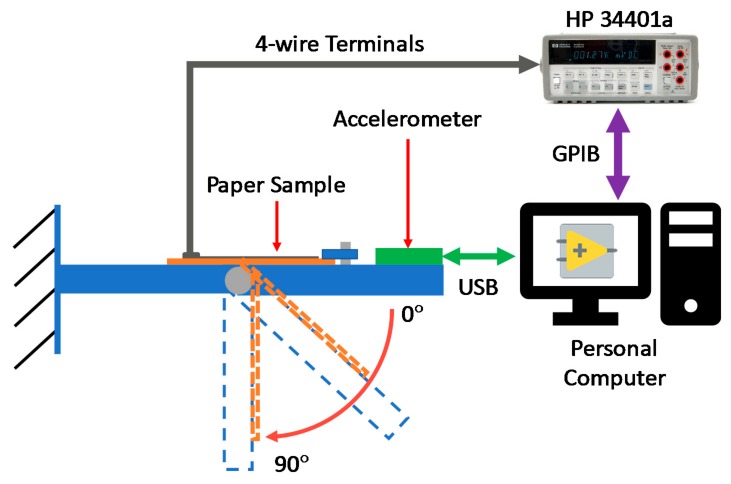
Schematic representation of the bending test.

A bending test was performed using a custom support fabricated via additive manufacturing, comprising a beam with a rotating part. Thus, it was possible to perform complete 0–90° manually operated movements. Resistance changings were recorded using the digital bench-top multimeter Hewlett-Packard 34401a (Agilent, Santa Clara, CA, USA) connected to a GPIB-USB (National Instruments, Austin, TX, USA) cable to a personal computer and monitored using a program written in LabVIEW. The bending angle measurement was simultaneously performed via the STEVAL-MKI005V1 MEMS (STMicroelctronics, Plan-les-Ouates, Geneva, Switzerland). Figure 8 shows a schematic representation of the experimental set-up employed during bending test.

**Figure 9:**
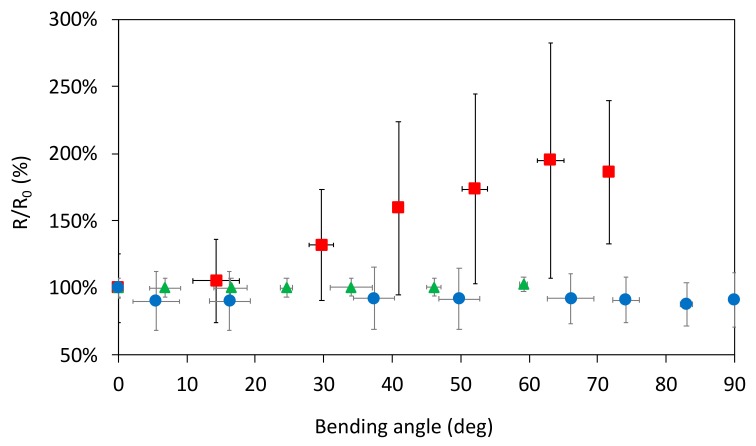
Resistance variations as a function of the bending angle on chromatographic paper (blue circle), cardboard (red square), and photographic paper (green triangle). Vertical error bars are related to standard deviation of resistance values, while horizontal ones are related to standard deviation of resistances values, while horizontal ones are related to standard deviation of angle values.

**Figure 10:**
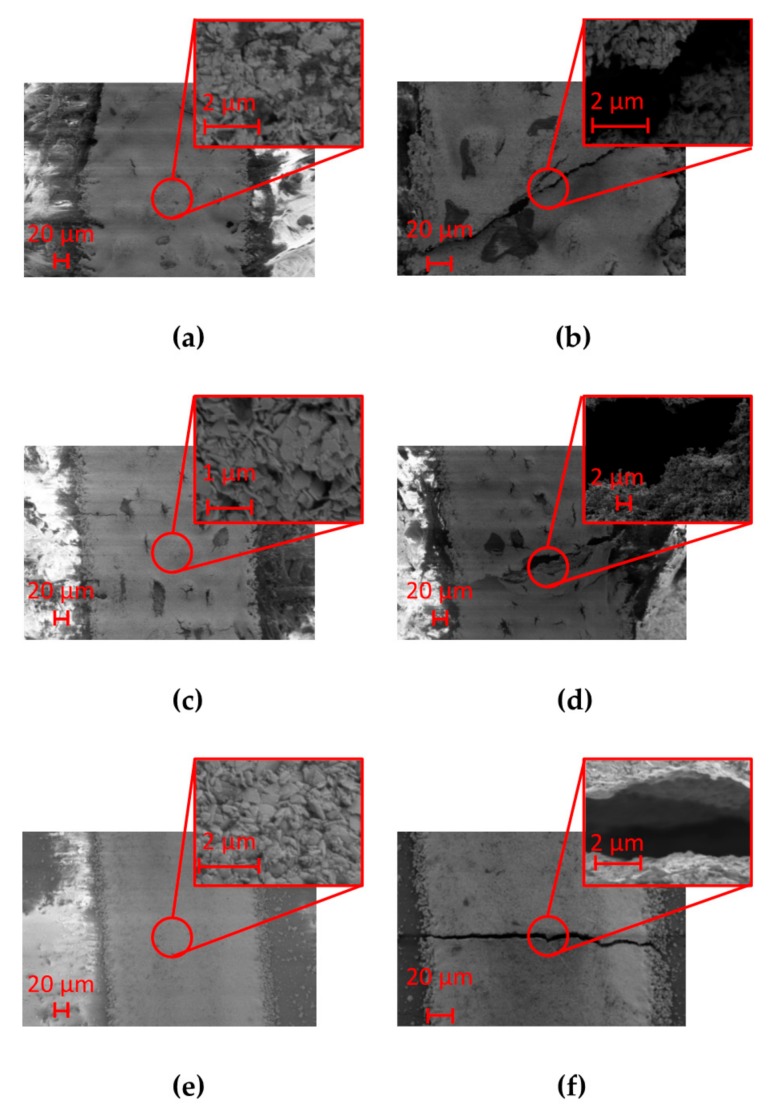
SEM magnifications of (**a**) chromatographic paper before bending, (**b**) chromatographic paper after bending, (**c**) cardboard before bending, (**d**) cardboard after bending, (**e**) photopaper before bending, (**f**) photopaper after bending.

Figure 9 shows how Ag ink samples paper behave under a bending load, also depending on the type of paper substrate. The results of these measurements are reported in terms of ratio between the resistance measured at a specific bending angle (R) versus its own resting resistance (R_0_). Considering chromatographic paper, in the range 0–90° only restricted variations take place with a quasi-stationary value, thus aiming that during the bending cycle the sample did not break up. Focusing the attention on cardboard, Figure 8 underlines that a substrate with a higher mechanical stiffness (with respect to chromatographic paper) shows limited variations until 70°, while reaching 80° the resistance value is about five times the starting value. Then possible cracks appear along the track and this fact led to an increase of orders of magnitude of resistance reaching a complete break up approaching to a bending angle of 90°. If compared to the other two substrates, photographic paper behaves in a third different way: the samples presented a quasi-constant value of resistance until 60°, during this first load time interval some cracks developed so that the samples undergo breaking before reaching the maximum degree of bending. To give evidence of these behaviors, SEM analysis was performed in order to state the microscopic aspect of the printed tracks before and after bending test Figure 10. Considering the samples on photopaper in Figure 10e,f and cardboard in Figure 10c,d, SEM magnifications evidenced the presence of severe cracks in Figure 10d,f, that caused variations in the section of the tracks until a total break up. SEM showed the presence of small cracks also on the surface of Ag tracks printed on chromatographic paper as shown in Figure 10b. Since we do not encounter any critical rupture in chromatographic samples, we assume that the influence of these cracks is superficial. Future studies that requires more samples and a different configuration of the setup will further analyze deeply this behavior.

### 3.3. Electrical Stability in Damp Environment

Ag samples printed on cellulose-based materials were also tested in a damp environment in order to check possible resistance variations because of an increased relative humidity (RH). Six samples, for each kind of substrate, were placed inside the incubator MCO-170M (PHC Panasonic Healthcare Holdings Co., Tokyo, Japan) in order to keep the temperature stable during the tests at around 27 °C (±0.2 °C). The damp atmosphere was generated because of an ultrasonic mist maker, temperature and relative humidity parameters were monitored because of the evaluation board HDC 1080 EVM (Texas Instruments, Dallas, TX, USA). Resistance changings were recorded for 13 min using the digital bench-top multimeter Hewlett-Packard 34401a controlled through a GPIB-USB cable by a custom-made LabVIEW Virtual Instruments interface.

Table 3 shows these measurements in terms of ratio between the sample resistance (R) measured at 35, 45, 55, 65, and 80% RH versus the sample resistance at the starting relative humidity point (R_35_). As expected, no significant resistance variation was registered during this test. These combinations of substrate and Ag ink can carefully work in presence of high relative humidity.

### 3.4. Multilayer Smart-3d-Device

**Figure 11:**
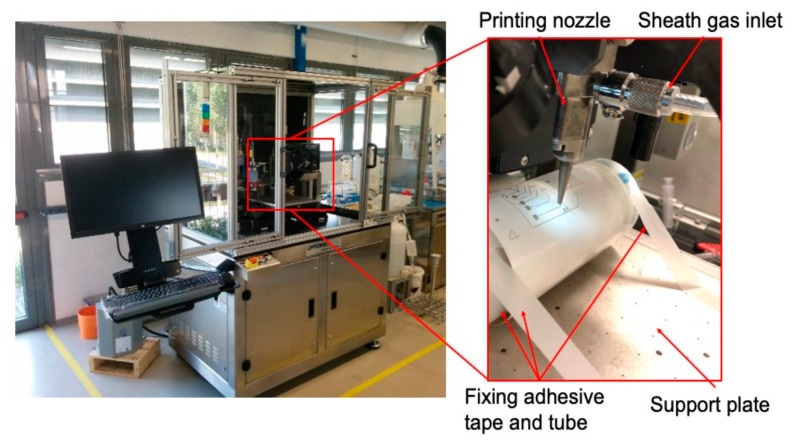
Optomec AJ300 with photopaper tube during printing step.

**Figure 12:**
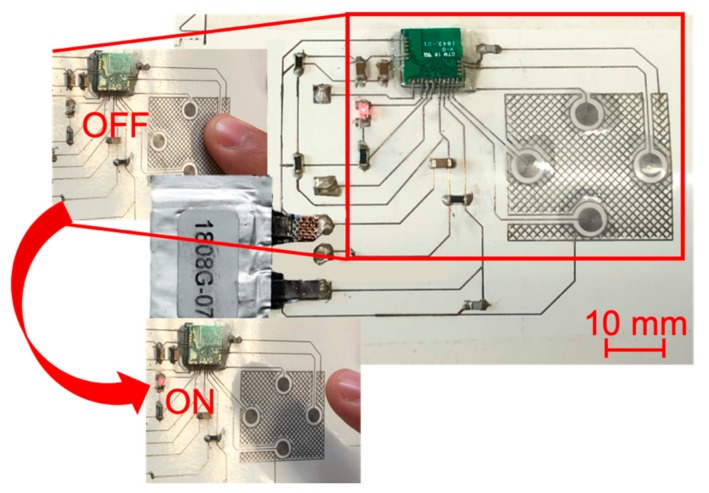
Multilayer smart-3D-device.

The proposed manufacturing method was used for the fabrication of a multilayer smart-3D-device. We fabricated the circuit through the proposed method on a photopaper tube Figure 11. The device basically consists of a touchpad made of four capacitive touch sensors, a LED and a microcontroller, which perform a simple function; while one capacitive touch sensors is pressed by the finger, the LED is kept turned off Figure 12 and touchpad data are transmitted via the integrated Bluetooth module. The microcontroller is Cypress CYBLE-222014-01 module; PSoC Creator was the selected software to configure the firmware of the microcontroller. A flexible battery of 3.7 V and 12 mAh was used to give power to the device (Lionrock Batteries, Kowloon, Hong Kong). Resistances (5.1 and 1.1 kΩ), capacitors (150 and 3.3 pF), a ferrite chip (330 Ω at 100 MHz), Cypress CYBLE-222014-01 module and a red LED were placed on the substrate, glued by means of NOA 81 and connected to the tracks. The abovementioned components are characterized by 0603 package.

**Figure 13:**
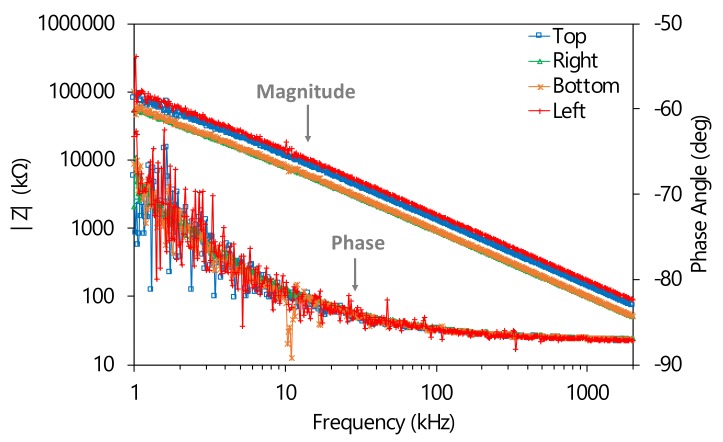
Impedance magnitude and phase angle plots for top, right, bottom, and left keys in no-finger state.

The tests on the printed capacitive touch sensors were realized both in case of pressing and not pressing the finger for ten times, by means of the impedance analyzer HP 4194A (Hewlett-Packard, Palo Alto, California, USA). Impedance/phase measurements between ground and each one of the four tracks of the capacitive sensors were performed from 1 kHz to 2 MHz using a compensated claw probe connected to analyzer HP4194A. Furthermore, electrical capacitance measurements were performed at 250 kHz, which is the working frequency of Cypress CYBLE-222014-01 module, by HP 4194A in fixed frequency configuration. Figure 13 shows impedance magnitude and phase angle of the four capacitive touch sensors, evidencing that in the analyzed range they have a purely capacitive behavior. Table 4 shows the capacitance values (average and standard deviation) of the four capacitive touch sensors in the two analyzed conditions. The data show an increase in electrical capacity of about 42% from no-finger to finger situation.

## 4. Conclusions

In this paper, we have reported a methodology to realize multilayer smart-3D-devices by means of Aerosol Jet Printing and Flash Lamp Annealing on cellulose-based substrates, specifically on chromatographic paper, photopaper, and cardboard. Curing parameters were optimized for each substrate, 1800 μs and 500 V for chromatographic paper, 1400 μs and 540 V for photopaper, 1200 μs and 560 V for cardboard. Optical microscope, profilometer, and SEM investigations have shown three different behaviors for the substrates in terms of line width and thickness. Under bending loads, chromatographic paper samples did not show any significant resistance variation suggesting they can still work properly, unlike sample printed on the other substrates that increase their resistance reaching a final break up. In presence of high relative humidity, silver tracks maintain their resistance values without any significant increase or decrease. The final circuit developed on photopaper tube is characterized by four capacitive touch sensors that if pressed, they switch off a LED. Electrical capacity measurements evidenced an increase of about 42% from non-pressed to pressed situation. The obtained results allow to demonstrate the applicability of the proposed method for the manufacture of smart electronic devices also on 3D cellulose substrates. Future studies will focus on the possibility of decreasing the variability in the processes to allow integration of sensors for other physical and chemical quantities. Furthermore, future studies on the possibilities of designing complex 3D geometries can lead to the evaluation of the fabrication limits of the proposed method. Then, the applicability of the proposed method in industrial field requires further investigation; in this research, these technologies were used in a laboratory environment, therefore, the adoption of the proposed manufacturing method in the industrial sector for the production of a large number of devices requires an integration of the two technologies (Aerosol Jet Printing and Flash Lamp Annealing) in one machine and the automation of the processes so as to allow a reduction of manual operations and fabrication time.

## Figures and Tables

**Table 1:** Printing process for Ag ink.

Process Parameters	HPS 108-AE1
Sheath gas flow (SCCM)	500
Atomizer gas flow (SCCM)	1350
Exhaust gas flow (SCCM)	1000
Plate temperature (°C)	35
Process speed (mm/s)	1

**Table 2:** Printing process for the photopolymer NOA 81.

Process Parameters	NOA 81
Sheath gas flow (SCCM)	600
Atomizer gas flow (SCCM)	1350
Exhaust gas flow (SCCM)	1000
Plate temperature (°C)	/
Process speed (mm/s)	1
Number of depositions	4

**Table 3:** Samples resistance behavior in a damp environment.

%RH	R/R_35_ (%)		
	Chromatographic Paper	Cardboard	Photopaper
35	100 ± 6	100 ± 4	100 ± 9
45	101 ± 9	95.3 ± 6	99.3 ± 8
55	102 ± 8	102 ± 6	99.7 ± 9
65	103 ± 6	95.7 ± 3	98.6 ± 8
80	96 ± 7	94.4 ± 8	97.3 ± 8

**Table 4:** Electrical capacitance values at 250 kHz for the capacitive sensors in case of finger in pression and not in pression.

Key Position	Capacitance (pF)	
	No Finger	Finger in Touch
Top	1.32 ± 0.08	1.86 ± 0.19
Right	1.92 ± 0.08	2.85 ± 0.35
Bottom	1.87 ± 0.09	2.72 ± 0.21
Left	1.13 ± 0.09	1.55 ± 0.15

## References

[1] Liu J., Yang C., Wu H., Lin Z., Zhang Z., Wang R., Li B., Kang F., Shi L., Wong C.P. (2014). Future paper based printed circuit boards for green electronics: Fabrication and life cycle assessment. Energy Environ. Sci..

[2] Chang H., Guo R., Sun Z., Wang H., Hou Y., Wang Q., Rao W., Liu J. (2018). Direct Writing and Repairable Paper Flexible Electronics Using Nickel–Liquid Metal Ink. Adv. Mater. Interfaces.

[3] Zou X., Chen C., Liang T., Xie J., Gillette-Henao E., Oh J., Tumalle J., Mazzeo A.D. (2018). Paper-Based Resistive Networks for Scalable Skin-Like Sensing. Adv. Electron. Mater..

[4] Wang Z., Fu X., Zhang Z., Jiang Y., Waqar M., Xie P., Bi K., Liu Y., Yin X., Fan R. (2019). Paper-based metasurface: Turning waste-paper into a solution for electromagnetic pollution. J. Clean. Prod..

[5] Bezuidenhout P., Smith S., Land K., Joubert T.H. (2019). Inkjet-printed interconnects for unpackaged dies in printed electronics. Electron. Lett..

[6] Guo Z., Jiao Y., Wang H., Zhang C., Liang F., Liu J.L., Yu H.D., Li C., Zhu G., Wang Z.L. (2019). Self-Powered Electrowetting Valve for Instantaneous and Simultaneous Actuation of Paper-Based Microfluidic Assays. Adv. Funct. Mater..

[7] Hu J., Wang S., Wang L., Li F., Pingguan-Murphy B., Lu T.J., Xu F. (2014). Advances in paper-based point-of-care diagnostics. Biosens. Bioelectron..

[8] Sun X., Wang H., Jian Y., Lan F., Zhang L., Liu H., Ge S., Yu J. (2018). Ultrasensitive microfluidic paper-based electrochemical/visual biosensor based on spherical-like cerium dioxide catalyst for miR-21 detection. Biosens. Bioelectron..

[9] Hayat A., Marty J.L. (2014). Disposable screen printed electrochemical sensors: Tools for environmental monitoring. Sensors.

[10] Tee-Ngam P., Nunant N., Rattanarat P., Siangproh W., Chailapakul O. (2013). Simple and rapid determination of ferulic acid levels in food and cosmetic samples using paper-based platforms. Sensors.

[11] Bezuidenhout P., Smith S., Joubert T.H. (2018). A low-cost inkjet-printed paper-based potentiostat. Appl. Sci..

[12] Wang Y., Guo H., Chen J.J., Sowade E., Wang Y., Liang K., Marcus K., Baumann R.R., Feng Z.S. (2016). Paper-Based Inkjet-Printed Flexible Electronic Circuits. ACS Appl. Mater. Interfaces.

[13] Lee C.J., Chang Y.C., Wang L.W., Wang Y.H. (2019). Biodegradable Materials for Organic Field-Effect Transistors on a Paper Substrate. IEEE Electron. Device Lett..

[14] Xie M.Z., Wang L.F., Dong L., Deng W.J., Huang Q.A. (2019). Low Cost Paper-Based LC Wireless Humidity Sensors and Distance-Insensitive Readout System. IEEE Sens. J..

[15] Shamkhalichenar H., Choi J.W. (2017). An inkjet-printed non-enzymatic hydrogen peroxide sensor on paper. J. Electrochem. Soc..

[16] Cinti S., Colozza N., Cacciotti I., Moscone D., Polomoshnov M., Sowade E., Baumann R.R., Arduini F. (2018). Electroanalysis moves towards paper-based printed electronics: Carbon black nanomodified inkjet-printed sensor for ascorbic acid detection as a case study. Sens. Actuators B Chem..

[17] Klem M.d.S., Morais R.M., Rubira R.J.G., Alves N. (2019). Paper-based supercapacitor with screen-printed poly (3, 4-ethylene dioxythiophene)-poly (styrene sulfonate)/multiwall carbon nanotube films actuating both as electrodes and current collectors. Thin Solid Films.

[18] Chinnadayyala S.R., Park J., Le H.T.N., Santhosh M., Kadam A.N., Cho S. (2019). Recent advances in microfluidic paper-based electrochemiluminescence analytical devices for point-of-care testing applications. Biosens. Bioelectron..

[19] Kit W., Olarnwanich A., Sriprachuabwong C., Karuwan C., Tuantranont A., Wisitsoraat A., Srituravanich W., Pimpin A. (2012). Disposable paper-based electrochemical sensor utilizing inkjet-printed Polyaniline modified screen-printed carbon electrode for Ascorbic acid detection. J. Electroanal. Chem..

[20] Tonello S., Abate G., Borghetti M., Marziano M., Serpelloni M., Uberti D.L., Lopomo N.F., Memo M., Sardini E. (2017). Wireless Point-of-Care Platform with Screen-Printed Sensors for Biomarkers Detection. IEEE Trans. Instrum. Meas..

[21] Borghetti M., Sardini E., Serpelloni M. Preliminary study of resistive sensors in inkjet technology for force measurements in biomedical applications. Proceedings of the 2014 IEEE 11th International Multi-Conference on Systems, Signals and Devices, SSD 2014.

[22] Borghetti M., Ghittorelli M., Sardini E., Serpelloni M., Torricelli F. (2016). Electrical characterization of PEDOT: PSS strips deposited by inkjet printing on plastic foil for sensor manufacturing. IEEE Trans. Instrum. Meas..

[23] Jo Y., Jeong D.W., Lee J.O., Choi Y., Jeong S. (2018). 3D-printed origami electronics using percolative conductors. RSC Adv..

[24] Ren’ai L., Zhang K., Chen G., Su B., Tian J., He M., Lu F. (2018). Green polymerizable deep eutectic solvent (PDES) type conductive paper for origami 3D circuits. Chem. Commun..

[25] Turner N. (2016). A Review of Origami and its Applications in Mechanical Engineering. Inst. Mech. Eng. Part C J. Mech. Eng. Sci..

[26] Linghu C., Zhang S., Wang C., Song J. (2018). Transfer printing techniques for flexible and stretchable inorganic electronics. NPJ Flex. Electron..

[27] Bian J., Zhou L., Wan X., Zhu C., Yang B., Huang Y. (2019). Laser Transfer, Printing, and Assembly Techniques for Flexible Electronics. Adv. Electron. Mater..

[28] Luo H., Wang C., Linghu C., Yu K., Wang C., Song J. (2019). Laser-Driven Programmable Non-Contact Transfer Printing of Objects onto Arbitrary Receivers via an Active Elastomeric Micro-Structured Stamp. Natl. Sci. Rev..

[29] Huang Q., Zhu Y. (2019). Printing Conductive Nanomaterials for Flexible and Stretchable Electronics: A Review of Materials, Processes, and Applications. Adv. Mater. Technol..

[30] Tan H.W., Tran T., Chua C.K. (2016). A review of printed passive electronic components through fully additive manufacturing methods. Virtual Phys. Prototyp..

[31] Hoey J.M., Lutfurakhmanov A., Schulz D.L., Akhatov I.S. (2012). A review on aerosol-based direct-write and its applications for microelectronics. J. Nanotechnol..

[32] Binder S., Glatthaar M., Rädlein E. (2014). Analytical investigation of aerosol jet printing. Aerosol Sci. Technol..

[33] OPTOMEC (2018). Aerosol Jet ® Printed Electronics Overview.

[34] Gupta A.A., Arunachalam S., Cloutier S.G., Izquierdo R. (2018). Fully Aerosol-Jet Printed, High-Performance Nanoporous ZnO Ultraviolet Photodetectors. ACS Photonics.

[35] Agarwala S., Goh G.L., Le T.D., An J., Peh Z.K., Yeong W.Y., Kim Y. (2019). Wearable Bandage-Based Strain Sensor for Home Healthcare: Combining 3D Aerosol Jet Printing and Laser Sintering. ACS Sens..

[36] Wang C., Hong G.Y., Li K.M., Young H.T. (2017). A miniaturized nickel oxide thermistor via aerosol jet technology. Sensors.

[37] Yang H., Rahman M.T., Du D., Panat R., Lin Y. (2016). 3-D printed adjustable microelectrode arrays for electrochemical sensing and biosensing. Sens. Actuators B Chem..

[38] Habermehl A., Strobel N., Eckstein R., Bolse N., Mertens A., Hernandez-Sosa G., Eschenbaum C., Lemmer U. (2017). Lab-on-chip, surface-enhanced Raman analysis by aerosol jet printing and roll-to-roll hot embossing. Sensors.

[39] Cantù E., Tonello S., Abate G., Uberti D., Sardini E., Serpelloni M. (2018). Aerosol jet printed 3D electrochemical sensors for protein detection. Sensors.

[40] Di Novo N.G., Cantù E., Tonello S., Sardini E., Serpelloni M. (2019). Support-material-free microfluidics on an electrochemical sensors platform by aerosol jet printing. Sensors.

[41] Park B.G., Lee C.J., Jung S.B. (2018). Enhancing adhesion strength of photonic sintered screen-printed Ag circuit by atmospheric pressure plasma. Microelectron. Eng..

[42] West J., Carter M., Smith S., Sears J. (2010). Photonic Curing of Silver Nanoparticle Based Inks. TechConnect Briefs.

[43] Das S., Cormier D., Williams S. (2015). Potential for Multi-functional Additive Manufacturing Using Pulsed Photonic Sintering. Procedia Manuf..

[44] Mudgal T., Bhadrachalam K., Bischoff P., Cormier D., Manley R.G., Hirschman K.D. (2017). Communication—CMOS Thin-Film Transistors via Xe Flash-Lamp Crystallization of Patterned Amorphous Si. ECS J. Solid State Sci. Technol..

[45] Schube J., Fellmeth T., Maier F., Keding R. (2018). Applicability of photonic sintering and autoclaving as alternative contact formation methods for silicon solar cells with passivating contacts Applicability of Photonic Sintering and Autoclaving as Alternative Contact Formation Methods for Silicon Solar C. AIP Conf. Proc..

[46] Bobinger M., Haider M., Goliya Y., Albrecht A., Becherer M., Lugli P., Rivadeneyra A., Russer J.A. (2018). On the sintering of solution-based silver nanoparticle thin-films for sprayed and flexible antennas. Nanotechnology.

[47] Akbari M., He H., Juuti J., Tentzeris M.M., Virkki J., Ukkonen L. (2017). 3D Printed and Photonically Cured Graphene UHF RFID Tags on Textile, Wood, and Cardboard Substrates. Int. J. Antennas Propag..

[48] Rizwan M., Kutty A.A., Kgwadi M., Drysdale T.D., Sydänheimo L., Ukkonen L., Virkki J. (2017). Possibilities of Fabricating Copper-Based RFID Tags with Photonic-Sintered Inkjet Printing and Thermal Transfer Printing. IEEE Antennas Wirel. Propag. Lett..

[49] Cronin H.M., Stoeva Z., Brown M., Shkunov M., Silva S.R.P. (2018). Photonic Curing of Low-Cost Aqueous Silver Flake Inks for Printed Conductors with Increased Yield. ACS Appl. Mater. Interfaces.

[50] Mahajan B.K., Ludwig B., Shou W., Yu X., Fregene E., Xu H., Pan H., Huang X. (2018). Aerosol printing and photonic sintering of bioresorbable zinc nanoparticle ink for transient electronics manufacturing. Sci. China Inf. Sci..

[51] Mahajan B.K., Yu X., Shou W., Pan H., Huang X. (2017). Mechanically Milled Irregular Zinc Nanoparticles for Printable Bioresorbable Electronics. Small.

